# Patterns and outcomes of health-care associated infections in the medical wards at Bugando medical centre: a longitudinal cohort study

**DOI:** 10.1186/s13756-023-01345-6

**Published:** 2023-12-05

**Authors:** Maliha I. Kassam, Vitus Silago, Prisca Damiano, Bahati Wajanga, Jeremiah Seni, Stephen E. Mshana, Samuel Kalluvya

**Affiliations:** 1 Department of Internal Medicine, Weill Bugando School of Medicine, Catholic University of Health and Allied Sciences, P. O. Box 1464, Mwanza, Tanzania; 2 Department of Internal Medicine, Bugando Medical Center, P. O. Box 1370, Mwanza, Tanzania; 3 Department of Microbiology and Immunology, Weill Bugando School of Medicine, Catholic University of Health and Allied Sciences, P. O. Box 1464, Mwanza, Tanzania

**Keywords:** Catheter-associated urinary tract Infection, Central-line-associated bloodstream Infection, Healthcare-associated Infections, Inpatients, Medical wards

## Abstract

**Background:**

The burden of healthcare associated infections (HCAIs) in low- and middle-income countries (LMICs) remains underestimated due to diagnostic complexity and lack of quality surveillance systems. We designed this study to determine clinical diagnosis, laboratory-confirmed, associated factors and risks of HCAIs.

**Methods:**

This hospital-based longitudinal cohort study was conducted between March and June 2022 among adults (≥ 18 years) admitted in medical wards at BMC in Mwanza, Tanzania. Patients who were negative for HCAIs by clinical evaluations and laboratory investigations during admission were enrolled and followed-up until discharge or death. Clinical samples were collected from patients with clinical diagnosis of HCAIs for conventional culture and antimicrobial sensitivity testing.

**Results:**

A total of 350 adult patients with a median [IQR] age of 54 [38–68] years were enrolled in the study. Males accounted for 54.6% (n = 191). The prevalence of clinically diagnosed HCAIs was 8.6% (30/350) of which 26.7% (8/30) had laboratory-confirmed HCAIs by a positive culture. Central-line-associated bloodstream infection (43.3%; 13/30) and catheter-associated urinary tract infection (36.7%; 11/30) were the most common HCAIs. Older age was the only factor associated with development of HCAIs [mean (± SD); [95%CI]: 58.9(± 12.5); [54.2–63.5] vs. 51.5(± 19.1); [49.4–53.6] years; p = 0.0391) and HCAIs increased the length of hospital stay [mean (± SD); [95%CI]: 13.8 (± 3.4); [12.5–15.1] vs. 4.5 (± 1.7); [4.3–4.7] days; p < 0.0001].

**Conclusion:**

We observed a low prevalence of HCAIs among adult patients admitted to medical wards in our setting. Central-line-associated bloodstream infections and catheter-associated urinary tract infections are common HCAIs. Significantly, older patients are at higher risk of acquiring HCAIs as well as patients with HCAIs had long duration of hospital stays.

## Introduction

Healthcare-associated infections (HCAIs) have a profound and far-reaching impact on patients and healthcare systems. These infections occur during medical treatment and can result in longer hospital stays, greater morbidity and mortality, and significant financial burdens [1]. For patients, HCAIs can lead to poor health outcomes, longer recovery periods, and sometimes even permanent disabilities [2]. For healthcare facilities, HCAIs strain resources and increase costs [3]. HCAIs require additional treatments, isolation measures, and sometimes readmission, which can escalate the financial burden on both patients and healthcare providers [4]. Therefore, preventing HCAIs is essential and can be achieved through rigorous infection control measures, hand hygiene, and antibiotic stewardship [5, 6].

The burden of HCAI is higher in low- and middle-income countries (LMICs) than high-income countries (HICs) [7, 8]. For instance, almost 7% of patients in HCIs and 10% of patients in LMICs will acquire at least one HCAI. Additionally, surgical site infection (SSI) is the commonest type of HCAIs and most prevalent accounting for 31% of all HCAIs reported among hospitalized patients [8]. However, the magnitude of HCAIs in LMICs remain underestimated because of the complexity in diagnosis and lack of quality surveillance system which requires expertise and resources [9]. Furthermore, overcrowding of patients and understaffing of healthcare professionals have led to poor infection prevention and control strategies, exacerbating the magnitude of HCAIs in LMICs [9].

In our setting, the prevalence of clinically diagnosed SSIs after caesarean section and major surgical procedures was 10.9% and 26.0% respectively [10, 11]. About 72.0% and 86.2% of the clinically diagnosed SSI after caesarean section and major surgical procedures were laboratory confirmed by aerobic culture. Generally, *Staphylococcus aureus*, *Escherichia coli* and *Klebsiella pneumoniae* were frequently isolated [10, 11]. The proportion of 18.8% of methicillin resistant *S. aureus* (MRSA) and 13.0% of extended spectrum beta-lactamase production among Gram-negative bacteria (ESBL-GNB) was reported among SSI after major surgeries [10]. About 80.0% of *K. pneumoniae* and *E. coli* were ESBL producers isolated from SSI after caesarean Sect. [11]. Pre-morbid illness, using iodine alone for skin preparation, prolonged duration of operation, and operation performed by junior surgeon and surgical wound class III were among risk factors leading to development of SSIs [10, 11].

Despite well-documented HCAIs, such as SSIs in surgical wards, little is known among patients admitted to medical wards. Consequently, hindering the implementation of specific preventive measures as well as timely and appropriately management of patients. This may facilitate spreading of bacterial pathogens and increase the associated morbidity and mortality. Therefore, this study aimed at investigating of clinical diagnosis and laboratory confirmed (implicating bacterial pathogens and antimicrobial susceptibility patterns), associated factors and risks of HCAIs among patients admitted in medical wards at Bugando Medical Centre (BMC) in Mwanza, Tanzania.

## Materials and methods

### Study design, duration, population and setting

This hospital-based longitudinal cohort study was conducted between March and June 2022 among adults (≥ 18 years) admitted in medical wards at BMC in Mwanza, Tanzania. BMC serves as teaching, consultancy and referral hospital for the Lake Zone regions (Mwanza, Shinyanga, Mara, Geita, Simiyu, Kagera and Kigoma) with 950 beds. Medical department at BMC has 188 beds distributed in private wing (n = 38), general male ward (n = 70), general female ward (n = 70) and adult intensive care unit (AICU; n = 10). A minimum sample size of 311 patients was calculated by [n = (Z_α/2_ +Z_β_)^2^ × (p1 (1 - p1) + p2 (1 - p2) ÷ (p1 - p2)^2^] at confidence level of 90% and power of 80%, α = 0.05, β = 0.2, p1 = 5.86% and p2 = 2.1% [12].

### Patients screening, enrollment and follow-up

All patients who consented to participate in the current study were screened for markers of infectious diseases such as full blood count and urinalysis at admission. This was done to rule out patients with subclinical infections before 48 h of admissions. The definition and screening of patients with clinical diagnosis of HCAIs based on previously documented protocol by HALT-3 (healthcare-associated infection in long-term care facilities) version 2.1 [13]. Other criteria for clinical diagnosis of HCAI were: all clinical symptoms and signs had to be new, non-infectious causes of signs and symptoms were excluded, suspicious of HCAI was not based on a single evidence as previously reported by Eilers et al., in 2012 [14]. In the current study, screening for HCAIs included clinical evaluation i.e., vital signs (body temperature, pulse rate, respiration rate and blood pressure) and laboratory investigations i.e., full blood count and urinalysis. Patients with normal (or negative) findings/results on screening tests were enrolled in the study and then followed-up on daily basis from 48 h after admission until final outcome (hospital discharge or death). Pre-tested structured questionnaire was used for collection of socio-demographic and clinical data whereas checklist was used during patient follow-up. However, after discharge patients were contacted by phone after every 7 days within 1 month to rule out if they develop HCAIs after hospital discharge. During follow-up, all patients who developed clinical presentation of HCAIs, the appropriate specimens e.g., urine for UTI-HCAI and blood for BSI-HCAI, were collected for aerobic bacteriological culture and antimicrobial susceptibility testing of isolated bacterial pathogens.

### Culture, biochemical identification, and antibiotic susceptibility testing

#### Culture

Respective clinical specimens from patients with clinical diagnosed patients of HCAIs were processed for implicating bacterial pathogens by conventional culture methods in Microbiology research laboratory at Catholic University of Health and Allied Sciences (CUHAS). Samples were directly inoculated on solid culture media (i.e., 5% sheep blood agar, chocolate agar, MacConkey agar, and Sabouraud dextrose agar; HiMedia, Maharashtra, India) except for blood samples which were firstly inoculated into brain heart infusion (BHI) broth by 1:10 and incubated for 24 h before being inoculated onto solid culture media [15, 16]. Negative blood cultures were monitored for 5 days before a final report [17]. Furthermore, urine samples were quantitatively inoculated onto solid culture media within 2 h after collection to rule out possible microbial contaminations [15, 16]. All incubations were done in ambient air at 35 ± 2 ^0^C for 24–48 h.

### Biochemical identification testing

Microbes’ colony morphology and characteristics on culture media were recorded and then Gram staining was performed to group bacteria into two major groups, Gram-positive cocci and Gram-negative rods, as well as Gram-positive budding yeast prior to biochemical identification testing (of bacteria) as previous documented [18]. Gram-negative rods were tested for sugar fermentation, CO_2_ and sulfur production on triple sugar ion agar; sulfur and indole production, and motility on sulfur-indole-motility agar; utilization of sodium citrate as the sole source of carbohydrate on Simmons citrate agar; and production of urease enzyme on Christensen’s urea agar. Gram-positive cocci were tested for catalase, coagulase and DNAse production; and sensitivity towards novobiocin and bacitracin; and hydrolysis of aesculin in presence of bile on bile aesculin agar. The media used for biochemical testing and identification of bacteria were from HiMedia, Maharashtra, India.

### Antibiotic susceptibility testing (AST) and phenotypic detection of ESBL-GNB and MRSA

Disk diffusion method by Kirby-Bauer technique [19] was used for antibiotic susceptibility testing. Pure and fresh bacterial colonies were suspended in sterile 0.85% physiological saline and the turbidity of the suspension was adjusted to 0.5McFarland on Densi-CHECK device (bioMérieux, SA). Bacterial suspensions were inoculated on entire surfaces of Mueller Hinton agar (MHA; HiMedia, Maharashtra, India) plates, then antibiotic disks were seeded within 15 min. All antibiotic disks used by the laboratory were produced by HiMedia, Maharashtra, India. Ampicillin (AMP 10 µg), trimethoprim-sulfamethoxazole (STX 1.25/23.75 µg), ceftriaxone (CRO 30 µg), ceftriaxone-sulbactam (CIS 30/15µg) ciprofloxacin (CIP 5 µg), gentamicin (GEN 10 µg), nitrofurantoin (NIT 300 units; for urine isolates only), amikacin (AK 10 µg) and meropenem (MEM 10 µg) were tested against Gram-negative bacteria (GNB). Whereas, STX 1.25/23.75 µg, CIP 5 µg, GEN 10 µg, NIT 300 units, erythromycin (ERY 15 µg), clindamycin (CLI 2 µg) and linezolid (LZD 30 µg) were tested against Gram-positive bacteria (GPB). Inoculated and seeded plates of MHA were incubated aerobically at 37 ± 2℃ for 16–18 h. Zones of inhibitions around antimicrobial disks were measured in millimeter and interpreted to sensitive or intermediate or resistant by using cut-off values from Clinical and Laboratory Standards Institute (CLSI) 2022 [20]. However, in the current study we interpreted zones of inhibitions of CIS 30/15µg based on cut-off values for CRO 30 µg by CLSI 2022.

Strains of *S. aureus* with zone diameter of ≤ 21 mm around FOX 30 µg disk were interpreted as MRSA [20]. On the other hand, GNB with resistance towards ceftriaxone were phenotypically confirmed for ESBL production by examining a zone difference between CRO 30 µg and CIS 30/15µg disks. A zone difference of ≥ 5 mm was interpreted as ESBL phenotype. ESBL phenotypes were further confirmed for carriage of genes encoding for ESBL production however our findings were published previously [21].

### Definition of technical term

Laboratory confirmed HCAI refers to isolation of pathogenic bacteria from biological specimen (e.g., blood, urine and pus) following diagnosis of HCAI based on clinical presentations.

### Data management and analysis

Data were entered into Microsoft excel for cleaning and coding, then imported to STATA software version 15.0 for analysis. Categorical variables were presented in percentages and fractions while continuous variables were presented in median (interquartile range; IQR). Independent t-test was used to compare mean age and mean days of hospitalization between patients without and with clinical diagnosis of HCAIs. A p-value of < 0.05 at 95% confidence interval [95%CI] was considered statistically significant.

## Results

### Socio-demographic characteristics of study participants

A total of 350 patients admitted in medical wards at BMC between March and June 2022 were enrolled in this study. Generally, the median [interquartile range; IQR] age of enrolled patients was 54 [38–68] years. Males accounted for 54.6% (n = 191), 63.7% (n = 223) were married and 44.3% (n = 155) had attained certificates of secondary education. Of 350 patients, 30 (8.6%) developed clinical symptoms of HCAIs during the course of follow-up of which females accounted for 53.3% (n = 16) and were older (62 [56–67] years) than their counterpart (Table 1).

**Table 1:** Socio-demographic characteristics of study participants

Characteristics	Categories	HCAIs (n = 30)	Non-HCAIs (n = 320)	Overall (N = 350)
		n(%)	n(%)	n(%)
Median [IQR] age in years		62 [56–67]	52 [36.5–68]	54 [38–68]
Sex	Females	16 (53.3)	143 (44.7)	159 (45.4)
	Males	14 (46.7)	177 (55.3)	191 (54.6)
Marital status	Single	0 (0.0)	48 (15.0)	48 (13.7)
	Married	20 (66.7)	203 (63.4)	223 (63.7)
	Widowed/separated	10 (33.3)	69 (21.6)	79 (22.6)
Education level	Primary	12 (40.0)	76 (23.7)	88 (25.1)
	Secondary	14 (46.7)	141 (44.1)	155 (44.3)
	Tertiary	4 (13.3)	103 (32.2)	107 (29.9)

### Clinical characteristics of study participants

Out of 350 patients, 56 (16.0%) were referred from other health-care facilities and mostly from regional referral hospital (35.7%; 20/56). By mobility, 44.9% (157/350) of the overall patients were ambulant while 50.0% (15/30) of patients with clinical HCAIs were on wheelchairs. Hemiplegia 14.6% (n = 51), hematemesis 9.1% (n = 32) and epigastric pain 7.1% (n = 25) were generally the commonest complaints for the current hospital admission. Almost all patients 99.4% (348/350) had invasive medical devices of which the majority had intra-vascular (IV) lines 93.4% (325/348) followed by urinary tract catheters 34.5% (120/348). Stroke was the commonest underlying disease condition among enrolled patients 20.5% (n = 73). The median [IQR] duration of hospital stay was 4 [3 - 6] days and the shortest stay was 2 days while the longest stay was 24 days. However, patients with clinical HCAIs stayed longer (13.5 [12 - 15] days) than patients without HCAIs. Of 350 patients, 12.3% (n = 43) died in the hospital (Table 2).

**Table 2:** Clinical characteristics of study participants

Characteristics	Categories	HCAIs (n = 30)	Non-HCAIs (n = 320)	Overall (N = 350)
		n(%)	n(%)	n(%)
Referral case	Yes	0 (0.0)	56 (17.5)	56 (16.0)
	No	30 (100)	264 (82.5)	294 (84.0)
Referring healthcare facility	Sekou Toure RRH	NA	20 (35.7)	20 (35.7)
	Geita RRH	NA	17 (30.3)	17 (30.3)
	Musoma RRH	NA	10 (17.9)	10 (17.9)
	Kitete	NA	7 (12.5)	7 (12.5)
	Kamanga Hospital	NA	1 (1.8)	1 (1.8)
	Bunda	NA	1 (1.8)	1 (1.8)
Mobility at admission	Bedridden	10 (33.3)	49 (15.3)	59 (16.9)
	Wheelchair	15 (50.0)	119 (37.2)	134 (38.3)
	Ambulant	5 (16.7)	152 (47.5)	157 (44.9)
Chief complain during current hospital admission	Hemiplegia	4 (13.3)	47 (14.7)	51 (14.6)
	Hematemesis	2 (6.7)	30 (9.3)	32 (9.1)
	Epigastric pain	2 (6.7)	23 (7.2)	25 (7.1)
	GBW	3 (10.0)	40 (12.5)	43 (12.3)
	DIB + anasarca	2 (6.7)	18 (5.6)	20 (5.7)
	Confusion	4 (13.3)	15 (4.7)	19 (5.4)
	Anuria	2 (6.7)	14 (4.4)	16 (4.5)
	Chest pain	1 (3.3)	13 (4.1)	14 (4.0)
	Joint pain	1 (3.3)	13 (4.1)	14 (4.0)
	Aphasia	3 (10.0)	9 (2.8)	12 (3.4)
	Palpations	0 (0.0)	10 (3.1)	10 (2.9)
	Others*	6 (20.0)	88 (27.5)	94 (26.9)
Presence of invasive device	No	0 (0.0)	2 (0.6)	2 (0.6)
	Yes	30 (100)	318 (99.4)	348 (99.4)
Type of invasive device	IV line	24 (80.0)	301 (94.6)	325 (93.4)
	Urinary tract catheter	14 (46.7)	106 (33.3)	120 (34.5)
	Hemodialysis catheter	6 (20.0)	17 (5.3)	23 (6.6)
	Nasogastric tube	0 (0.0)	2 (0.6)	2 (0.5)
Underlying disease conditions	Stroke	7 (23.3)	66 (20.6)	73 (20.9)
	UGIB	2 (6.7)	24 (7.5)	26 (7.4)
	CCF + DCM	2 (6.7)	20 (6.3)	22 (6.3)
	ESRD	0 (0.0)	22 (6.9)	22 (6.3)
	Gastritis	1 (3.3)	17 (5.3)	18 (5.1)
	CCF	1 (3.3)	16 (5.0)	17 (4.9)
	HTN emergency	2 (6.7)	14 (4.4)	16 (4.6)
	Anaemia	2 (6.7)	13 (4.1)	15 (4.3)
	VOC + SCD	1 (3.3)	13 (4.1)	14 (4.0)
	DKA + T2DM	1 (3.3)	9 (2.8)	10 (2.9)
	Others**	11 (36.7)	106 (33.1)	117 (33.3)
Median [IQR] days of hospital stay		13.5 [12–15]	4 [3–5]	4 [3–6]
Hospital discharge status	Died	2 (6.7)	41 (12.8)	43 (12.3)
	Alive	28 (93.3)	279 (87.2)	307 (87.7)

**KEY:** CCF = congestive cardiac failure; GBW = generalized body weakness; DCM = dilated cardiomyopathy; DIB = difficulty in breathing; DKA = diabetic ketoacidosis; ESRD = end-stage renal disease; HTN = hypertension; NGT = nasogastric tube; UGIB = upper gastrointestinal bleeding; RRH = regional referral hospital; SCD = sickle cell disease; T2DM = type 2 diabetes mellitus; RTI = respiratory tract infection; VOC = vaso-occlusive crisis

Others*: ascites, blurry vision + headache, convulsion, dizziness, hematochezia, hemoptysis, melena, lower limb edema, paroxysmal nocturnal dyspnea, oliguria, and polyuria

Others**: rheumatic heart disease, asthma, cancer associated conditions, arrhythmia, colitis, epilepsy, cardiogenic shock, hepatitis B infection + liver cirrhosis, intestinal mass, migraine, cardiomyopathy, thyrotoxicosis, uremia, and unstable angina

### Prevalence and types of laboratory confirmed HCAIs and descriptions of patients with HCAIs

The overall prevalence of clinically diagnosed HCAIs is 8.6% (30/350) of which CLABSI (43.3%; 13/30) and CAUTI (36.7%; 11/30) were frequently diagnosed. The median [IQR] age of patients with clinical diagnosis of HCAIs was 62 [56–67] years. The median [IQR] duration of hospital stay from admission to development of HCAIs was 7.5 [7 - 10] days while the median [IQR] duration of hospital stay for patients with HCAIs was 13.5 [12 - 15] days. Females accounted for 53.3% (n = 16) among patients with clinical diagnosis of HCAIs (Table 3). To note, no patient developed infection after hospital discharge during the whole time of follow-up.

The prevalence of laboratory confirmed HCAIs among patients with clinical HCAIs is 26.7% (8/30) of which 62.5% (5/8) were CAUTI, 25.0% (2/8) were CLABSI and 12.5% (1/8) was RTI. Two patients died of which 1 had clinical CLABSI and another had laboratory confirmed CAUTI (Tables 3 and 4).

**Table 3:** Clinical diagnosis and laboratory confirmed HCAI among study participants

Variables		Frequency (n)	Percentages (%)
Median [IQR] days to development of HCAI		7.5 [7–10]	-
Types of clinical HCAIs diagnosed	CLABSI	13	43.3
	CAUTI	11	36.7
	Others*	6	20.0
On antibiotic use during sample collection	Yes	8	26.6
	No	22	73.3
Laboratory confirmed HCAI	Yes	8	26.7
	No	22	73.3

**KEY:** CAUSTI = catheter-associated urinary tract infection and CLABSI = central-line-associated blood-stream infection

Others*: enteritis (n = 1), respiratory tract infection (n = 1), sepsis (n = 1) and central nervous system infection (n = 1)

**Table 4:** Descriptions of patients with clinical and laboratory diagnosis of HCAIs admitted in medical wards at BMC

Sex	Age	Mobility	Reason for admission	Invasive device	Underlying disease	Clinical HCAI	Antibiotic use	Lab culture results	Organism isolated	Length	Status
Male	47	Bedridden	Dyspnoea	Canula, urinary catheter	CCF, DCM	CLABSI	Ceftriaxone	Negative	NA	19	Alive
Female	63	Wheelchair	Lower back pain	Canula, urinary catheter	MM	CAUTI	Metronidazole	Negative	NA	15	Alive
Female	58	Ambulant	Epigastric pain	Canula, urinary catheter	PUD	CAUTI	Metronidazole	Negative	NA	8	Alive
Female	58	Wheelchair	Hemiplegia	Canula, urinary catheter	Stroke	CAUTI	No	Negative	NA	15	Alive
Male	62	Bedridden	Hemiplegia	Canula, urinary catheter	Stroke	CLABSI	No	Negative	NA	20	Alive
Female	70	Ambulant	GBW	Canula	Anaemia	Enteritis	No	Negative	NA	16	Alive
Female	68	Wheelchair	DIB, Anasarca	Canula, urinary catheter	CCF	CAUTI	No	Positive	*E. coli* and *P. aeruginosa*	13	Alive
Female	63	Wheelchair	Anuria	Hemodialysis catheter	ESRD	CLABSI	No	Positive	*S. aureus*	14	Alive
Male	78	Bedridden	Aphasia	Canula, urinary catheter	Stroke	CAUTI	No	Negative	NA	17	Alive
Male	56	Ambulant	Hematemesis	Canula	UGIB, VARICES	CAUTI	No	Negative	NA	12	Alive
Male	80	Bedridden	Paraplegia	Canula, urinary catheter	MM	CAUTI	No	Positive	*Acinetobacter* spp	13	Alive
Male	59	Wheelchair	Jaundice	Canula	HCC, PHTN, HEP B	RTI	No	Positive	*S. pyogenes*	15	Alive
Male	62	Wheelchair	Confusion	Hemodialysis catheter	ESRD	CLABSI	No	Negative	NA	15	Alive
Male	59	Bedridden	Abdominal distention	Canula	PHTN	CLABSI	No	Negative	NA	14	Alive
Female	50	Wheelchair	Headache	Canula	HTN	CLABSI	No	Negative	NA	11	Alive
Female	38	Wheelchair	Confusion	Hemodialysis catheter	Electrolyte imbalance	CLABSI	Cirofloxacin	Negative	NA	13	Died
Female	65	Bedridden	GBW	Canula, urinary catheter	DKA, T2DM	Sepsis	Ceftriaxone	Negative	NA	13	Alive
Male	68	Bedridden	Hemiplegia	Canula, urinary catheter	Stroke	Sepsis	No	Negative	NA	11	Alive
Male	72	Wheelchair	GBW	Canula	Anaemia	CLABSI	Ceftriaxone	Negative	NA	24	Alive
Female	42	Wheelchair	Confusion	Hemodialysis catheter	Uremia	CLABSI	No	Negative	NA	14	Alive
Female	62	Wheelchair	Chest pain	Canula	ACS	Sepsis	Azithromycin	Negative	NA	15	Alive
Male	58	Bedridden	DIB, Anasarca	Canula, urinary catheter	CCF	CAUTI	No	Positive	*Candida* spp	16	Alive
Female	62	Bedridden	Aphasia	Canula, urinary catheter	Stroke	CAUTI	No	Positive	*E. coli*	11	Died
Female	25	Ambulant	Joints pain	Canula	VOC, SCD	CNSI	No	Negative	NA	14	Alive
Male	35	Wheelchair	Hematemesis	Canula	UGIB, VARICES	CLABSI	Ciprofloxacin	Negative	NA	12	Alive
Female	45	Ambulant	Epigastric pain	Canula	NON	CLABSI	No	Negative	NA	7	Alive
Female	64	Wheelchair	Confusion	Hemodialysis catheter	ESRD	CLABSI	No	Negative	NA	13	Alive
Male	72	Wheelchair	Aphasia	Canula, urinary catheter	Stroke	CAUTI	No	Negative	NA	11	Alive
Female	67	Bedridden	Hemiplegia	Canula, urinary catheter	Stroke	CAUTI	No	Positive	*E. coli*	11	Alive
Male	58	Wheelchair	Anuria	Hemodialysis catheter	ESRD	CLABSI	No	Positive	*S. aureus*	12	Alive

**KEY:** GBW = generalized body weakness; CAUSTI = catheter-associated urinary tract infection; CCF = congestive cardiac failure; CLABSI = central-line-associated blood-stream infection; CNSI = central nervous system infection; DCM = dilated cardiomyopathy; DIB = difficulty in breathing; DKA = diabetic ketoacidosis; ESRD = end-stage renal disease; HCC = hepatocellular carcinoma; HEP B = hepatitis B; HTN = hypertension; MM = multiple myeloma; NA = not applicable; PHTN = pulmonary hypertension; PUD = peptic ulcer disease; T2DM = type 2 diabetes mellitus; UGIB = upper gastrointestinal bleeding; and RTI = respiratory tract infection

### Antibiotic susceptibility patterns of bacteria causing HCAIs among patients admitted in medical wards at BMC

Generally, all GNB were resistant to ampicillin, trimethoprim-sulfamethoxazole, ceftriaxone, and ciprofloxacin. However, all GNB bacteria were susceptible towards ceftriaxone-sulbactam, meropenem, and amikacin. Two among five and three among four of the GNB were susceptible to gentamicin and nitrofurantoin respectively. *E. coli* (n = 3) and *Acinetobacter* spp., (n = 1) showed resistance to ceftriaxone but were susceptible to ceftriaxone-sulbactam. On the other hand, all GPB were resistant to trimethoprim-sulfamethoxazole, ciprofloxacin, and gentamicin while they were all susceptible to linezolid. Furthermore, 1/3 and 2/3 of GPB were susceptible to erythromycin and clindamycin respectively. One *S. aureus* was found to be methicillin resistant *S. aureus* (MRSA) strain. Overall, 5 (62.5%) out of 8 patients with laboratory confirmed HCAIs had infection with multidrug resistant bacteria strains i.e., ESBL or MRSA (Table 5).

**Table 5:** Antibiotic susceptibility patterns of bacteria causing HCAIs among patients admitted in medical wards at BMC

Isolate	AMP	SXT	CRO	CIS	CIP	GEN	NIT	MEM	AK	CLI	ERY	LZD	FOX
*E. coli*	R	R	R	S	R	R	S	S	S	NA	NA	NA	NA
*E. coli*	R	R	R	S	R	S	S	S	S	NA	NA	NA	NA
*E. coli*	R	R	R	S	R	S	S	S	S	NA	NA	NA	NA
*Acinetobacter* spp.,	NA	R	R	S	R	R	R	S	S	NA	NA	NA	NA
*P. aeruginosa*	NA	NA	NA	NA	R	R	NA	S	S	NA	NA	NA	NA
*S. aureus*	NT	R	NA	NA	R	I	NA	NA	NA	S	S	S	S
*S. aureus*	NT	R	NA	NA	R	R	NA	NA	NA	R	R	S	R
*S. pyogenes*	NT	R	NA	NA	I	I	NA	NA	NA	S	R	S	NA
*Candida* spp.	NA	NA	NA	NA	NA	NA	NA	NA	NA	NA	NA	NA	NA

**KEY:** AK = amikacin; AMP = ampicillin; CIP = ciprofloxacin; CIS = ceftriaxone-sulbactam; CLI = clindamycin; CRO = ceftriaxone; ERY = erythromycin; FOX = cefoxitin; GEN = gentamicin; I = intermediate; LZD = linezolid; MEM = meropenem; NA = not applicable; NIT = nitrofurantoin; NT = not tested; R = resistant; S = susceptible; and SXT = trimethoprim-sulfamethoxazole

### Determinant and impact of HCAIs among patients admitted in medical wards at BMC

By independent t-test analysis, increased patient age [mean (± SD): 58.9(± 12.5) vs. 51.5(± 19.1) years, p = 0.0391] was the only determinant for development of HCAI among patients admitted in medical wards/units. On the other hand, HCAI was associated with increased days of hospitalization [mean (± SD): 13.8 (± 3.4) vs. 4.5 (± 1.7) days, p < 0.001] (Table 5). The length of hospital stay (mean (± SD); [95%CI]) was 5.5 (± 3.3) [4.3–6.6] days between clinical diagnosis of HCAIs and outcome (death or discharge) among patients with HCAIs.

**Table 6:** Determinant and impact of HCAIs among patients admitted in medical wards at BMC

Variable	Patients without HCAI		Patients with HCAI		Mean difference	p-value
	**Mean (± SD)**	**95%CI**	**Mean(± SD)**	**95%CI**		
Age in years	51.5(± 19.1)	[49.4–53.6]	58.9(± 12.5)	[54.2–63.5]	− 7.4	0.0391
Hospital stay in days	4.5(± 1.7)	[4.3–4.7]	13.8(± 3.4)	[12.5–15.1]	− 9.3	< 0.0001

**KEY:** CI = confidence interval; HCAI = health-care associated infection; and SD = standard deviation

## Discussion

The current study examined the patterns and outcomes of healthcare associated infections (HCAIs) among adult inpatients admitted in medical wards at a zonal referral hospital in North-western of Tanzania. The majority of patients enrolled had advanced age and were self-referrals. Nearly one half of patients were ambulant during hospital admissions. On the other hand, almost all patients had invasive medical devices which included intra-venous (IV) lines for administration of fluids and medications [22] and urinary catheters for draining of urine [23]. Generally, by average patients’ length of hospital stay was 4 days although the shortest stay was 2 days while the longest stay was 24 days. The prevalence of clinical and laboratory confirmed HCAIs among adult inpatients in medical wards at our setting is low. About 12.3% of enrolled patients died in the hospital during the course of medical care.

We document a low prevalence (8.5%) of clinical HCAIs among adult patients admitted in medical wards at our setting. CLBSI (43.3%) and CAUTI (36.7%) were the commonest HCAIs observed. Low prevalence of clinical HCAIs was also reported previously from similar settings [24, 25]. For instance, a study in Kenya by Patil et al., in 2022 reported that 41 out of 952 patient cases developed HCAIs [24]. Another study in Ethiopia by Taye et al., in 2023 reported a prevalence of 8.88% [25]. In line with our findings, both studies from Kenya and Ethiopia reported that bloodstream infection, respiratory tract infection, urinary tract infection and gastroenteritis are commonest HCAIs. In the current study, most of patients with clinical HCAIs were females, on wheelchair by mobility, had medical invasive devices with IV-line and urinary catheters being frequent encountered medical devices. Moreover, nearly one quarter of patients with clinical HCAIs had stroke as an underlying condition as well as eight patients were on empiric antibiotic therapy during sample collection.

The prevalence of laboratory confirmed HCAIs in the current study is also low (26.7%; 8/30) as compared to a study from Kenya by Patil and colleagues who reported that 25 out of 41 patient cases had a positive culture [24]. The fact that Patil and colleagues in Kenya enrolled patients aged less than 18 years, included patients in surgical wards, and included anaerobic culture as well as detection of viral pathogens unlikely our study may be the reason why they observed high prevalence of laboratory confirmed HCAIs [24]. In the current study, we isolated 9 pathogens from 8 positive cultures. The pathogens included *E. coli*, *P. aeruginosa*, *Acinetobacter* spp., and *Candida* spp., isolated from patients with CAUTI; *S. aureus* isolated from patients with CLABSI; and *S. pyogenes* isolated from patient with RTI. We performed antimicrobial susceptibility testing for bacteria pathogens and observed that generally all bacteria exhibited resistance towards multiple antibiotic agents tested. Furthermore, *E. coli* (n = 2), *Acinetobacter* spp., (n = 1) and *P. aeruginosa* (n = 1) were characterized for carriage of genes encoding for ESBL production and found that *E. coli* and *P. aeruginosa* were carrying *bla*_CTX−M_ and *bla*_TEM_ genes [21]. Given the small sample size of enrolled patients and subsequent small number of patients confirmed with HCAI, we are recommending a long term prospective study in the AMR surveillance context so that generated findings can be extrapolated and used to inform changes in the treatment guidelines at our setting.

Two patients with diagnosis of HCAIs died in the hospital in the current study. The first patient was female, aged 38 years and on dialysis with hemodialysis catheter as an indwelling medical device. Additionally, this patient had electrolyte imbalance and confusion. Later on, the patient developed clinical presentations of CLABSI and was put on ciprofloxacin however her blood culture turned out negative. A growing body of literature documented that dialysis as a result of kidney disease and electrolytes imbalance increases the risk of BSI [26, 27]. A combination of electrolyte imbalance and BSI accelerates patient’s condition towards confusion and death [28–30]. Exposure to antibiotic i.e., ciprofloxacin prior to blood sample collection may be linked with a negative blood culture as observed in our case and as documented previously [31]. The second patient was also female, aged 62 years, bedridden by mobility and had aphasia due to stroke. This patient had IV-line and urinary tract catheter as indwelling medical devices. Later on the patient developed clinical presentations of CAUTI which was later confirmed by a positive urine culture with the isolation of ESBL producing *E. coli*. Indwelling urinary catheterization notably prolonged use of the urinary catheter is documented to be the most important risk factor for developing CAUTI [32]. Therefore, basic practices for prevention of CAUTI as previously documented by Lo and colleagues are warranted [33].

Furthermore, we observed that older patients are at higher risk of acquiring HCAIs as well as HCAIs were associated with increased days of hospitalization. Similar findings that the risk of HCAIs increases linearly with age were reported previously [34, 35]. This may be explained by the fact that, as the immune system ages (due to increasing in age), the immune function declines resulting to a condition known as immunosenescence [36, 37]. Together with age related organ changes, comorbidities, malnutrition and polypharmacy, the normal capabilities of defense against infectious diseases decline [36, 38]. On the other account, in line with previous studies [39, 40] we found that HCAIs was associated with increased days of hospitalization. We also observed an increased days of hospitalization attributable to HCAIs as compared to overall days of hospitalization among study participants (5.5 (± 3.3) vs. 4.5 (± 1.7) days). This can be due to the fact that, occurrence of HCAI is associated with additional days in patient’s management hence prolonging days of stay in healthcare facilities.

### Limitations

The current study was limited by small sample size, lack of anaerobic culture and detection of other infectious agents such as viruses which may underestimated the prevalence of laboratory confirmed HCAIs.

## Conclusion

We document low prevalence of HCAIs among adult patients admitted in medical wards at our setting. Central-line-associated bloodstream infection and catheter-associated urinary tract infection are common HCAIs. Significantly, older patients are at higher risk of acquiring HCAIs as well as patients with HCAIs had long duration of hospital stays. A long term prospective study is recommended in the context of on-going AMR surveillance so that generated data can be used to inform local evidence-based treatment guidelines.

## Data Availability

The datasets used and/or analysed during the current study are available from the corresponding author on reasonable request.
